# An ortho­rhom­bic polymorph of 1-[(ferrocen­yl)(hydr­oxy)meth­yl]-1,2-dicarba-*closo*-dodecaborane

**DOI:** 10.1107/S1600536809051903

**Published:** 2009-12-09

**Authors:** Hongwei Chen, Jinling Miao, Peihua Zhu, Daqi Wang, Yong Nie

**Affiliations:** a School of Chemistry and Chemical Engineering, University of Jinan, Jinan 250022, People’s Republic of China; b College of Chemistry and Chemical Engineering, Liaocheng University, Liaocheng 252059, People’s Republic of China

## Abstract

An ortho­rhom­bic polymorph of the title compound, [Fe(C_5_H_5_)(C_8_H_17_B_10_O)] or C_13_H_22_B_10_FeO, is described here in addition to the known monoclinic polymorph [Crundwell *et al.* (1999 ▶). *Acta Cryst.* **C55**, IUC9900087]. The asymmetric unit contains four independent mol­ecules with C_cage_–C_cage_ distances of 1.636 (16)–1.700 (16) Å, and with the methyl­hydr­oxy groups disordered over two positions in each mol­ecule [occupancy ratios 0.80 (2):0.20 (2), 0.59 (3):0.41 (3), 0.60 (2):0.40 (2) and 0.793 (17):0.207 (17)].

## Related literature

For the crystal structure of the monoclinic polymorph, see Crundwell *et al.* (1999 ▶). For the crystal structures of related carboranyl alcohols, see: Tsuji (2004 ▶); Terrasson *et al.* (2008 ▶); Shen *et al.* (2006 ▶).
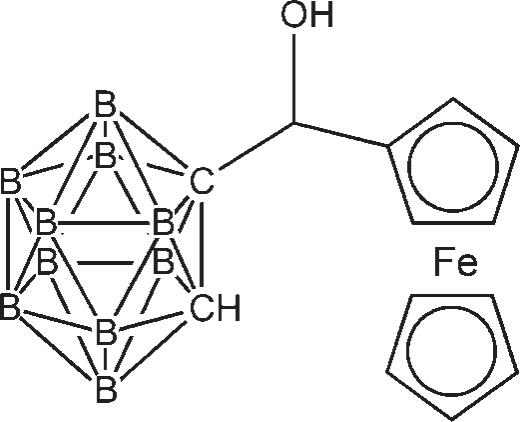


## Experimental

### 

#### Crystal data

[Fe(C_5_H_5_)(C_8_H_17_B_10_O)]
*M* *_r_* = 358.26
Orthorhombic, 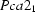

*a* = 27.051 (2) Å
*b* = 8.9682 (7) Å
*c* = 29.191 (3) Å
*V* = 7081.8 (11) Å^3^
*Z* = 16
Mo *K*α radiation
μ = 0.85 mm^−1^
*T* = 298 K
0.25 × 0.14 × 0.07 mm

#### Data collection

Bruker SMART CCD area-detector diffractometer
Absorption correction: multi-scan (*SADABS*; Bruker, 2001 ▶) *T*_min_ = 0.816, *T*_max_ = 0.943
35277 measured reflections
12025 independent reflections
5629 reflections with *I* > 2σ(*I*)
*R*_int_ = 0.115

#### Refinement

*R*[*F* ^2^ > 2σ(*F* ^2^)] = 0.075
*wR*(*F* ^2^) = 0.181
*S* = 1.03
12025 reflections
942 parameters
1 restraint
H-atom parameters constrained
Δρ_max_ = 1.12 e Å^−3^
Δρ_min_ = −0.45 e Å^−3^
Absolute structure: Flack (1983 ▶), 5617 Friedel pairs
Flack parameter: 0.02 (3)

### 

Data collection: *SMART* (Bruker, 2001 ▶); cell refinement: *SAINT* (Bruker, 2001 ▶); data reduction: *SAINT*; program(s) used to solve structure: *SHELXS97* (Sheldrick, 2008 ▶); program(s) used to refine structure: *SHELXL97* (Sheldrick, 2008 ▶); molecular graphics: *SHELXTL* (Sheldrick, 2008 ▶); software used to prepare material for publication: *SHELXTL*.

## Supplementary Material

Crystal structure: contains datablocks I, global. DOI: 10.1107/S1600536809051903/cv2664sup1.cif

Structure factors: contains datablocks I. DOI: 10.1107/S1600536809051903/cv2664Isup2.hkl

Additional supplementary materials:  crystallographic information; 3D view; checkCIF report

## Figures and Tables

**Fig. 1.**
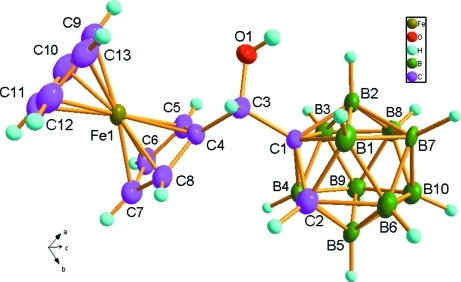
The molecular structure of one independent molecule of (I) showing the atomic numbering and 25% probability displacement ellipsoids. Only major parts of the disordered atoms are shown for clarity.

## supplementary crystallographic information

### Comment

In 1999 Crundwell *et al.* reported an interesting compound having an *o*-carboranyl and a ferrocenyl moieties tethered through a hydroxymethyl group, from the reaction of *o*-carborane and ferrocenecarboxaldehyde mediated by tetrabutylammonium fluoride. We have recently studied a similar reaction of dilithiocarborane with ferrocenecarboxaldehyde in the hope of obtaining a novel species that have two ferrocenyl groups linking to the carborane cage *via* a single carbon atom. It turned out that the reaction occurred only at one cage carbon yielding after recrystallization a new polymorph of the known compound (Crundwell *et al.*, 1999)

The title compound (I) crystallizes in the orthorhombic *Pca*2_1_ space group (*cf.* that of Crundwell *et al.* in the monoclinic *P*2_1_/*c* space group) with the normal geometric parameters comparable with those observed in related structures of carboranyl alcohols (Tsuji, 2004; Terrasson *et al.*, 2008; Shen *et al.*, 2006).

There are four crystallographically independent molecules in the asymmetric unit, and all the hydroxyl groups are disordered over two positions with different occupancy for each oxygen atom. These molecules have the same constitution and very similar geometric parameters, only some subtle differences are found within the carborane cage. The C_cage_—C_cage_ distances in the present structure vary in the range 1.636 (16)–1.700 (16) Å, while that of Crundwell *et al.* has been reported to be 1.658 (3) Å.

### Experimental

Under argon a portion of *n*-BuLi (2.2 *M*, 0.23 ml, 0.5 mmol) was added dropwise to a solution of *o*-carborane (36 mg, 0.25 mmol) in diethylether (20 ml) cooled with ice-bath. The resulting suspension was stirred at ambient temperature for 0.5 h, cooled to 253 K, to which ferrocenecarboxaldehyde (106 mg, 0.5 mmol) was added. The reaction mixture was warmed up to ambient temperature and stirred for 2 h. After quenching with HCl (10%) the mixture was filtered to remove small amount of black stuff and extracted with ether. The organic phases were combined and dried (MgSO_4_) to give a yellow solid, which was purified with preparative TLC (eluent: CH_2_Cl_2_/ petroleum ether (b.p. 333–363 K) (1:1, V/V). A yellow band at *Rf* = 0.51 was collected and identified to be the title compound (34 mg, 38%). Yellow crystals were grown from a dichloromethane/*n*-hexane solution at ambient temperature. m.p. 464–465 K. IR (KBr): *v* = 3552 (*w*), 3082 (*w*), 2896 (*w*), 2585 (*s*) cm^-1^.

### Refinement

All H-atoms were positioned geometrically (C—H  0.93-0.98 Å; B—H 1.1 Å; O—H 0.82 Å), and refined using a riding model, with *U*_iso_=1.2*U*_eq_ of the parent atom.

### Crystal data

**Table:**

[Fe(C_5_H_5_)(C_8_H_17_B_10_O)]	*D*_x_ = 1.344 Mg m^−^^3^
*M**_r_* = 358.26	Mo *K*α radiation, λ = 0.71073 Å
Orthorhombic, *P**c**a*2_1_	Cell parameters from 3704 reflections
*a* = 27.051 (2) Å	θ = 2.6–25.3°
*b* = 8.9682 (7) Å	µ = 0.85 mm^−^^1^
*c* = 29.191 (3) Å	*T* = 298 K
*V* = 7081.8 (11) Å^3^	Yellow, block
*Z* = 16	0.25 × 0.14 × 0.07 mm
*F*(000) = 2944	

### Data collection

**Table:**

Bruker SMART CCD area-detector diffractometer	12025 independent reflections
Radiation source: fine-focus sealed tube	5629 reflections with *I* > 2σ(*I*)
graphite	*R*_int_ = 0.115
phi and ω scans	θ_max_ = 25.0°, θ_min_ = 1.4°
Absorption correction: multi-scan (*SADABS*; Bruker, 2001)	*h* = −32→32
*T*_min_ = 0.816, *T*_max_ = 0.943	*k* = −10→9
35277 measured reflections	*l* = −34→33

### Refinement

**Table:**

Refinement on *F*^2^	Secondary atom site location: difference Fourier map
Least-squares matrix: full	Hydrogen site location: inferred from neighbouring sites
*R*[*F*^2^ > 2σ(*F*^2^)] = 0.075	H-atom parameters constrained
*wR*(*F*^2^) = 0.181	*w* = 1/[σ^2^(*F*_o_^2^) + (0.0599*P*)^2^] where *P* = (*F*_o_^2^ + 2*F*_c_^2^)/3
*S* = 1.03	(Δ/σ)_max_ = 0.001
12025 reflections	Δρ_max_ = 1.12 e Å^−^^3^
942 parameters	Δρ_min_ = −0.45 e Å^−^^3^
1 restraint	Absolute structure: Flack (1983), 5617 Friedel pairs
Primary atom site location: structure-invariant direct methods	Flack parameter: 0.02 (3)

### Special details

**Table:**

Geometry. All e.s.d.'s (except the e.s.d. in the dihedral angle between two l.s. planes) are estimated using the full covariance matrix. The cell e.s.d.'s are taken into account individually in the estimation of e.s.d.'s in distances, angles and torsion angles; correlations between e.s.d.'s in cell parameters are only used when they are defined by crystal symmetry. An approximate (isotropic) treatment of cell e.s.d.'s is used for estimating e.s.d.'s involving l.s. planes.
Refinement. Refinement of *F*^2^ against ALL reflections. The weighted *R*-factor *wR* and goodness of fit *S* are based on *F*^2^, conventional *R*-factors *R* are based on *F*, with *F* set to zero for negative *F*^2^. The threshold expression of *F*^2^ > σ(*F*^2^) is used only for calculating *R*-factors(gt) *etc*. and is not relevant to the choice of reflections for refinement. *R*-factors based on *F*^2^ are statistically about twice as large as those based on *F*, and *R*- factors based on ALL data will be even larger.

### Fractional atomic coordinates and isotropic or equivalent isotropic displacement parameters (Å^2^)

**Table:**

	*x*	*y*	*z*	*U*_iso_*/*U*_eq_	Occ. (<1)
Fe1	0.02406 (6)	0.5567 (2)	0.22116 (6)	0.0499 (5)	
Fe2	0.24220 (6)	0.43970 (18)	0.47382 (5)	0.0470 (4)	
Fe3	0.37998 (6)	0.94455 (19)	0.34605 (5)	0.0458 (4)	
Fe4	0.40150 (6)	0.93997 (19)	0.09112 (4)	0.0451 (4)	
O1	0.1333 (3)	0.6277 (13)	0.2864 (4)	0.078 (5)	0.80 (2)
H1	0.1623	0.6514	0.2896	0.116*	0.80 (2)
O2	0.3554 (5)	0.342 (2)	0.5263 (7)	0.076 (8)	0.59 (3)
H2	0.3728	0.3236	0.5487	0.115*	0.59 (3)
O3	0.2684 (5)	0.8410 (17)	0.2913 (5)	0.072 (6)	0.60 (2)
H3	0.2408	0.8042	0.2892	0.109*	0.60 (2)
O4	0.5111 (3)	0.8624 (11)	0.0271 (3)	0.069 (4)	0.793 (17)
H4	0.5398	0.8343	0.0251	0.103*	0.793 (17)
O1'	0.1373 (15)	0.729 (5)	0.2444 (18)	0.08 (2)	0.20 (2)
H1'	0.1635	0.7621	0.2543	0.116*	0.20 (2)
O2'	0.3530 (7)	0.241 (3)	0.4882 (9)	0.076 (11)	0.41 (3)
H2'	0.3740	0.1755	0.4909	0.115*	0.41 (3)
O3'	0.2719 (7)	0.728 (2)	0.3309 (8)	0.072 (10)	0.40 (2)
H3'	0.2456	0.6908	0.3226	0.108*	0.40 (2)
O4'	0.5154 (13)	0.746 (4)	0.0740 (13)	0.069 (16)	0.207 (17)
H4'	0.5427	0.7271	0.0632	0.103*	0.207 (17)
B1	0.1600 (5)	0.9955 (16)	0.2925 (5)	0.060 (4)	
H1A	0.1840	0.9835	0.2622	0.072*	
B2	0.1619 (5)	0.8823 (15)	0.3371 (5)	0.051 (4)	
H2A	0.1884	0.7893	0.3357	0.061*	
B3	0.1070 (5)	0.8518 (16)	0.3630 (4)	0.052 (4)	
H3A	0.0971	0.7441	0.3785	0.063*	
B4	0.0629 (5)	0.9635 (15)	0.3349 (4)	0.051 (4)	
H4A	0.0237	0.9325	0.3323	0.061*	
B5	0.0821 (5)	1.1460 (15)	0.3405 (5)	0.051 (4)	
H5	0.0551	1.2377	0.3407	0.061*	
B6	0.1416 (5)	1.1706 (17)	0.3159 (5)	0.060 (4)	
H6	0.1526	1.2783	0.3010	0.072*	
B7	0.1836 (4)	1.0591 (13)	0.3436 (4)	0.038 (3)	
H7	0.2228	1.0913	0.3461	0.046*	
B8	0.1517 (6)	0.9647 (18)	0.3890 (6)	0.060 (5)	
H8	0.1702	0.9339	0.4213	0.072*	
B9	0.0871 (7)	1.0227 (18)	0.3850 (6)	0.059 (5)	
H9	0.0628	1.0318	0.4151	0.071*	
B10	0.1363 (6)	1.1474 (16)	0.3759 (5)	0.057 (4)	
H10	0.1445	1.2367	0.4006	0.068*	
B11	0.3273 (5)	0.1532 (19)	0.6143 (5)	0.065 (4)	
H11	0.3195	0.2648	0.6281	0.078*	
B12	0.3791 (5)	0.1062 (18)	0.5876 (6)	0.063 (5)	
H12	0.4075	0.1929	0.5829	0.076*	
B13	0.3733 (6)	−0.0116 (19)	0.5468 (6)	0.064 (5)	
H13	0.3966	−0.0077	0.5160	0.077*	
B14	0.3114 (5)	−0.0589 (17)	0.5443 (5)	0.066 (4)	
H14	0.2930	−0.0887	0.5120	0.079*	
B15	0.2950 (6)	−0.1456 (18)	0.5955 (5)	0.069 (5)	
H15	0.2661	−0.2317	0.5962	0.083*	
B16	0.3056 (7)	−0.0143 (19)	0.6427 (6)	0.069 (5)	
H16	0.2840	−0.0171	0.6746	0.083*	
B17	0.3695 (7)	0.037 (2)	0.6398 (6)	0.073 (6)	
H17	0.3902	0.0745	0.6701	0.087*	
B18	0.3959 (5)	−0.0737 (18)	0.5999 (5)	0.061 (4)	
H18	0.4337	−0.1158	0.6048	0.074*	
B19	0.3545 (6)	−0.1780 (18)	0.5721 (5)	0.072 (5)	
H19	0.3647	−0.2876	0.5582	0.086*	
B20	0.3502 (6)	−0.1441 (18)	0.6307 (5)	0.063 (4)	
H20	0.3568	−0.2317	0.6564	0.076*	
B21	0.3462 (5)	0.5429 (15)	0.2318 (4)	0.054 (4)	
H21	0.3845	0.5832	0.2352	0.065*	
B22	0.3012 (5)	0.6486 (16)	0.2047 (4)	0.051 (4)	
H22	0.3095	0.7593	0.1904	0.061*	
B23	0.2456 (5)	0.6038 (15)	0.2302 (4)	0.049 (4)	
H23	0.2167	0.6898	0.2325	0.058*	
B24	0.2548 (6)	0.4790 (17)	0.2725 (5)	0.060 (4)	
H24	0.2318	0.4793	0.3036	0.072*	
B25	0.3321 (5)	0.3534 (14)	0.2237 (5)	0.057 (3)	
H25	0.3610	0.2677	0.2212	0.068*	
B26	0.3224 (6)	0.4866 (17)	0.1801 (5)	0.056 (4)	
H26	0.3453	0.4897	0.1490	0.068*	
B27	0.2572 (6)	0.5254 (18)	0.1781 (5)	0.055 (5)	
H27	0.2371	0.5529	0.1465	0.066*	
B28	0.2296 (5)	0.4210 (16)	0.2242 (6)	0.059 (4)	
H28	0.1912	0.3815	0.2232	0.071*	
B29	0.2773 (5)	0.3167 (18)	0.2495 (5)	0.064 (4)	
H29	0.2707	0.2058	0.2644	0.077*	
B30	0.2779 (5)	0.3421 (16)	0.1903 (5)	0.060 (4)	
H30	0.2711	0.2512	0.1657	0.071*	
B31	0.4803 (5)	0.6450 (16)	−0.0499 (4)	0.046 (3)	
H31	0.4686	0.7517	−0.0650	0.055*	
B32	0.4394 (5)	0.5353 (15)	−0.0195 (5)	0.053 (4)	
H32	0.4008	0.5710	−0.0149	0.063*	
B33	0.4709 (5)	0.4354 (15)	0.0232 (5)	0.046 (3)	
H33	0.4524	0.4052	0.0554	0.055*	
B34	0.5342 (5)	0.4931 (17)	0.0201 (5)	0.046 (4)	
H34	0.5578	0.5035	0.0506	0.056*	
B35	0.5585 (5)	0.4340 (15)	−0.0310 (5)	0.053 (4)	
H35	0.5978	0.4035	−0.0338	0.063*	
B36	0.5261 (6)	0.5250 (18)	−0.0765 (5)	0.052 (4)	
H36	0.5441	0.5532	−0.1092	0.062*	
B37	0.4638 (5)	0.4738 (18)	−0.0729 (5)	0.046 (4)	
H37	0.4405	0.4670	−0.1037	0.055*	
B38	0.4578 (5)	0.3433 (15)	−0.0269 (5)	0.048 (3)	
H38	0.4310	0.2512	−0.0281	0.058*	
B39	0.5153 (5)	0.3209 (16)	−0.0009 (4)	0.051 (4)	
H39	0.5259	0.2155	0.0157	0.062*	
B40	0.5120 (5)	0.3421 (15)	−0.0624 (4)	0.049 (4)	
H40	0.5207	0.2508	−0.0862	0.058*	
C1	0.1115 (4)	0.8805 (13)	0.3052 (4)	0.046 (3)	
C2	0.0972 (4)	1.0492 (14)	0.2886 (4)	0.067 (4)	
H2B	0.0803	1.0736	0.2551	0.080*	
C3	0.1063 (4)	0.7509 (13)	0.2709 (4)	0.059 (3)	
H3B	0.1200	0.7827	0.2414	0.070*	0.80 (2)
H3'B	0.1144	0.6706	0.2923	0.070*	0.20 (2)
C4	0.0537 (4)	0.7096 (13)	0.2640 (4)	0.052 (3)	
C5	0.0237 (5)	0.6067 (16)	0.2896 (5)	0.074 (4)	
H5A	0.0338	0.5441	0.3132	0.089*	
C6	−0.0245 (5)	0.6211 (17)	0.2709 (5)	0.081 (4)	
H6A	−0.0519	0.5681	0.2812	0.097*	
C7	−0.0255 (5)	0.7227 (15)	0.2361 (5)	0.080 (4)	
H7A	−0.0528	0.7501	0.2187	0.096*	
C8	0.0222 (4)	0.7769 (14)	0.2321 (4)	0.068 (4)	
H8A	0.0321	0.8486	0.2110	0.082*	
C9	0.0625 (6)	0.3699 (17)	0.2050 (5)	0.086 (5)	
H9A	0.0852	0.3195	0.2232	0.103*	
C10	0.0120 (7)	0.3459 (17)	0.2046 (6)	0.094 (6)	
H10A	−0.0056	0.2799	0.2230	0.112*	
C11	−0.0072 (7)	0.442 (2)	0.1708 (6)	0.097 (6)	
H11A	−0.0400	0.4505	0.1615	0.116*	
C12	0.0340 (8)	0.5224 (19)	0.1541 (6)	0.090 (5)	
H12A	0.0332	0.5962	0.1318	0.107*	
C13	0.0733 (7)	0.4752 (19)	0.1755 (6)	0.078 (5)	
H13A	0.1050	0.5116	0.1704	0.093*	
C14	0.3298 (4)	0.1115 (14)	0.5573 (4)	0.056 (3)	
C15	0.2808 (4)	0.0463 (14)	0.5878 (4)	0.070 (3)	
H15A	0.2427	0.0874	0.5848	0.084*	
C16	0.3235 (4)	0.2333 (15)	0.5206 (5)	0.068 (4)	
H16A	0.3312	0.1870	0.4910	0.081*	0.59 (3)
H16'	0.3368	0.3166	0.5385	0.081*	0.41 (3)
C17	0.2703 (4)	0.2884 (12)	0.5177 (4)	0.046 (3)	
C18	0.2486 (5)	0.4058 (15)	0.5425 (4)	0.067 (4)	
H18A	0.2642	0.4685	0.5634	0.081*	
C19	0.1984 (4)	0.4093 (15)	0.5294 (4)	0.067 (4)	
H19A	0.1751	0.4755	0.5410	0.080*	
C20	0.1885 (4)	0.2999 (15)	0.4968 (4)	0.068 (4)	
H20A	0.1584	0.2816	0.4825	0.081*	
C21	0.2328 (4)	0.2234 (13)	0.4898 (3)	0.058 (3)	
H21A	0.2372	0.1428	0.4702	0.069*	
C22	0.2819 (6)	0.6162 (17)	0.4530 (5)	0.075 (4)	
H22A	0.3053	0.6638	0.4711	0.090*	
C23	0.2919 (6)	0.4956 (16)	0.4239 (5)	0.067 (5)	
H23A	0.3226	0.4527	0.4186	0.080*	
C24	0.2489 (6)	0.4538 (17)	0.4052 (4)	0.069 (4)	
H24A	0.2442	0.3743	0.3853	0.083*	
C25	0.2126 (6)	0.5510 (19)	0.4210 (5)	0.077 (4)	
H25A	0.1795	0.5471	0.4126	0.092*	
C26	0.2327 (7)	0.6546 (17)	0.4511 (5)	0.082 (5)	
H26A	0.2167	0.7317	0.4663	0.098*	
C27	0.2975 (3)	0.6116 (12)	0.2615 (3)	0.043 (3)	
C28	0.3172 (4)	0.4385 (14)	0.2755 (4)	0.070 (4)	
H28A	0.3360	0.4120	0.3079	0.084*	
C29	0.3023 (4)	0.7313 (14)	0.2967 (4)	0.063 (3)	
H29A	0.2949	0.6842	0.3262	0.075*	0.60 (2)
H29'	0.2874	0.8128	0.2791	0.075*	0.40 (2)
C30	0.3527 (4)	0.7936 (12)	0.3009 (3)	0.048 (3)	
C31	0.3920 (4)	0.7303 (13)	0.3276 (4)	0.057 (3)	
H31A	0.3905	0.6478	0.3468	0.068*	
C32	0.4326 (5)	0.8194 (15)	0.3183 (4)	0.066 (4)	
H32A	0.4636	0.8044	0.3313	0.080*	
C33	0.4222 (5)	0.9325 (15)	0.2879 (4)	0.068 (4)	
H33A	0.4437	1.0054	0.2770	0.082*	
C34	0.3726 (4)	0.9135 (13)	0.2771 (4)	0.055 (3)	
H34A	0.3551	0.9729	0.2566	0.066*	
C35	0.3725 (6)	0.9481 (16)	0.4157 (4)	0.071 (4)	
H35A	0.3770	0.8674	0.4353	0.086*	
C36	0.4102 (6)	1.0491 (19)	0.4019 (5)	0.079 (5)	
H36A	0.4432	1.0485	0.4108	0.094*	
C37	0.3855 (6)	1.1561 (15)	0.3701 (5)	0.076 (4)	
H37A	0.4007	1.2336	0.3543	0.091*	
C38	0.3345 (5)	1.1179 (14)	0.3685 (4)	0.062 (4)	
H38A	0.3098	1.1686	0.3526	0.074*	
C39	0.3282 (6)	0.9884 (17)	0.3955 (5)	0.071 (5)	
H39A	0.2985	0.9376	0.3993	0.085*	
C40	0.4872 (4)	0.6142 (12)	0.0076 (3)	0.037 (3)	
C41	0.5375 (3)	0.6109 (12)	−0.0249 (4)	0.044 (3)	
H41	0.5644	0.7027	−0.0241	0.053*	
C42	0.4819 (4)	0.7461 (13)	0.0409 (4)	0.049 (3)	
H42	0.4939	0.7131	0.0709	0.059*	0.793 (17)
H42'	0.4935	0.8281	0.0215	0.059*	0.207 (17)
C43	0.4296 (4)	0.7875 (11)	0.0462 (3)	0.041 (2)	
C44	0.4042 (4)	0.9010 (14)	0.0229 (4)	0.057 (3)	
H44	0.4180	0.9624	0.0006	0.068*	
C45	0.3567 (5)	0.9086 (15)	0.0374 (4)	0.066 (4)	
H45	0.3333	0.9778	0.0278	0.079*	
C46	0.3491 (5)	0.7979 (14)	0.0684 (4)	0.068 (4)	
H46	0.3190	0.7753	0.0822	0.082*	
C47	0.3939 (4)	0.7229 (12)	0.0764 (4)	0.053 (3)	
H47	0.3993	0.6461	0.0972	0.064*	
C48	0.4458 (6)	1.1224 (15)	0.1078 (5)	0.075 (4)	
H48	0.4681	1.1734	0.0894	0.090*	
C49	0.3928 (6)	1.1530 (16)	0.1136 (5)	0.081 (5)	
H49	0.3748	1.2272	0.0989	0.097*	
C50	0.3733 (6)	1.0434 (18)	0.1474 (5)	0.077 (5)	
H50	0.3409	1.0354	0.1578	0.092*	
C51	0.4129 (6)	0.9562 (16)	0.1602 (4)	0.069 (4)	
H51	0.4110	0.8803	0.1818	0.083*	
C52	0.4555 (6)	0.9945 (16)	0.1372 (5)	0.069 (4)	
H52	0.4858	0.9463	0.1400	0.082*	

### Atomic displacement parameters (Å^2^)

**Table:**

	*U*^11^	*U*^22^	*U*^33^	*U*^12^	*U*^13^	*U*^23^
Fe1	0.0616 (11)	0.0438 (10)	0.0442 (9)	−0.0073 (9)	−0.0085 (8)	−0.0061 (10)
Fe2	0.0522 (10)	0.0455 (9)	0.0431 (9)	0.0043 (9)	−0.0062 (8)	0.0038 (10)
Fe3	0.0526 (9)	0.0439 (10)	0.0407 (9)	−0.0038 (9)	−0.0082 (7)	−0.0036 (10)
Fe4	0.0505 (10)	0.0439 (11)	0.0409 (9)	0.0075 (8)	0.0093 (8)	−0.0048 (10)
O1	0.051 (6)	0.077 (9)	0.105 (11)	0.014 (6)	−0.020 (5)	−0.029 (8)
O2	0.050 (9)	0.078 (13)	0.102 (15)	−0.013 (8)	−0.009 (8)	0.039 (12)
O3	0.047 (8)	0.072 (11)	0.097 (12)	0.002 (7)	−0.008 (7)	−0.031 (9)
O4	0.048 (6)	0.071 (8)	0.087 (8)	−0.014 (5)	0.019 (5)	−0.030 (6)
O1'	0.05 (3)	0.08 (3)	0.11 (4)	0.01 (2)	−0.02 (2)	−0.03 (3)
O2'	0.050 (13)	0.078 (18)	0.10 (2)	−0.013 (11)	−0.009 (12)	0.039 (17)
O3'	0.047 (12)	0.072 (17)	0.097 (19)	0.002 (10)	−0.008 (11)	−0.031 (14)
O4'	0.05 (2)	0.07 (3)	0.09 (3)	−0.014 (19)	0.02 (2)	−0.03 (2)
B1	0.054 (9)	0.058 (10)	0.067 (10)	−0.021 (8)	0.013 (8)	−0.012 (8)
B2	0.045 (8)	0.049 (8)	0.059 (9)	−0.005 (7)	−0.008 (7)	−0.020 (7)
B3	0.057 (9)	0.049 (9)	0.051 (8)	−0.006 (7)	0.006 (7)	−0.004 (7)
B4	0.043 (7)	0.053 (9)	0.057 (8)	−0.012 (7)	0.001 (6)	−0.010 (7)
B5	0.055 (8)	0.040 (8)	0.058 (9)	−0.008 (7)	−0.002 (7)	−0.015 (7)
B6	0.062 (9)	0.054 (10)	0.066 (10)	−0.017 (8)	0.001 (8)	−0.012 (8)
B7	0.037 (6)	0.031 (7)	0.046 (7)	−0.021 (6)	0.007 (6)	−0.010 (7)
B8	0.063 (11)	0.063 (11)	0.055 (9)	0.001 (9)	−0.009 (8)	−0.007 (8)
B9	0.057 (11)	0.056 (11)	0.063 (10)	−0.003 (8)	0.010 (9)	−0.012 (8)
B10	0.061 (10)	0.053 (10)	0.055 (9)	−0.008 (8)	0.003 (7)	−0.013 (7)
B11	0.066 (10)	0.065 (11)	0.065 (10)	0.009 (9)	−0.003 (8)	0.010 (8)
B12	0.056 (10)	0.068 (12)	0.066 (10)	0.011 (7)	0.008 (8)	0.018 (10)
B13	0.062 (10)	0.069 (11)	0.061 (10)	0.020 (8)	−0.005 (8)	0.018 (9)
B14	0.065 (9)	0.065 (11)	0.067 (10)	0.011 (8)	−0.008 (7)	0.004 (9)
B15	0.066 (10)	0.059 (10)	0.082 (12)	0.005 (8)	−0.006 (9)	0.021 (9)
B16	0.068 (11)	0.082 (13)	0.059 (10)	0.019 (9)	0.004 (8)	0.020 (9)
B17	0.070 (12)	0.079 (14)	0.069 (12)	0.009 (10)	−0.016 (9)	0.008 (10)
B18	0.050 (8)	0.068 (11)	0.066 (10)	0.023 (8)	−0.008 (8)	0.011 (8)
B19	0.073 (10)	0.064 (11)	0.078 (11)	0.022 (9)	−0.008 (9)	0.004 (9)
B20	0.063 (10)	0.063 (11)	0.063 (10)	0.012 (9)	−0.007 (8)	0.027 (8)
B21	0.046 (7)	0.061 (9)	0.054 (9)	0.001 (7)	0.002 (6)	−0.012 (7)
B22	0.051 (9)	0.048 (9)	0.055 (9)	−0.016 (7)	0.002 (7)	0.000 (7)
B23	0.034 (7)	0.054 (9)	0.057 (10)	−0.005 (7)	−0.005 (6)	−0.017 (7)
B24	0.049 (9)	0.072 (12)	0.059 (9)	−0.018 (8)	0.003 (7)	−0.009 (8)
B25	0.046 (7)	0.056 (8)	0.069 (9)	−0.005 (7)	−0.007 (7)	−0.013 (8)
B26	0.048 (9)	0.070 (11)	0.052 (8)	−0.013 (7)	0.009 (7)	−0.016 (7)
B27	0.056 (10)	0.061 (12)	0.048 (9)	0.002 (8)	−0.016 (7)	−0.002 (8)
B28	0.051 (9)	0.066 (10)	0.061 (9)	−0.012 (8)	−0.008 (7)	0.001 (9)
B29	0.061 (9)	0.063 (11)	0.069 (10)	−0.010 (8)	−0.016 (8)	0.009 (8)
B30	0.053 (9)	0.055 (10)	0.071 (10)	−0.001 (8)	−0.007 (7)	−0.011 (8)
B31	0.049 (8)	0.044 (9)	0.045 (8)	0.004 (7)	−0.002 (7)	0.002 (7)
B32	0.048 (8)	0.048 (9)	0.063 (9)	−0.003 (7)	0.005 (7)	−0.009 (8)
B33	0.048 (8)	0.043 (9)	0.045 (7)	0.002 (7)	0.003 (6)	−0.003 (7)
B34	0.043 (8)	0.049 (9)	0.047 (9)	0.009 (6)	−0.004 (6)	−0.009 (7)
B35	0.043 (7)	0.055 (9)	0.060 (9)	0.002 (7)	−0.001 (7)	−0.012 (8)
B36	0.056 (10)	0.049 (11)	0.050 (9)	−0.009 (7)	0.012 (7)	−0.011 (7)
B37	0.045 (9)	0.048 (11)	0.044 (8)	0.005 (7)	−0.005 (6)	−0.008 (7)
B38	0.051 (8)	0.040 (8)	0.053 (8)	−0.007 (6)	0.007 (7)	−0.011 (7)
B39	0.054 (9)	0.047 (9)	0.053 (8)	0.013 (7)	0.000 (7)	−0.006 (7)
B40	0.053 (9)	0.045 (9)	0.048 (8)	0.005 (7)	0.002 (6)	−0.010 (6)
C1	0.037 (6)	0.048 (7)	0.052 (7)	−0.004 (6)	0.001 (5)	−0.012 (6)
C2	0.072 (9)	0.061 (9)	0.067 (8)	−0.011 (7)	−0.002 (6)	−0.004 (7)
C3	0.044 (7)	0.060 (9)	0.072 (8)	−0.004 (6)	0.004 (7)	−0.020 (7)
C4	0.046 (7)	0.057 (8)	0.054 (7)	−0.005 (6)	−0.003 (6)	−0.005 (6)
C5	0.076 (10)	0.080 (11)	0.067 (9)	−0.013 (8)	0.001 (7)	−0.013 (8)
C6	0.063 (9)	0.095 (12)	0.085 (10)	−0.032 (8)	0.005 (8)	−0.023 (9)
C7	0.069 (9)	0.081 (10)	0.090 (11)	−0.001 (8)	−0.011 (8)	−0.019 (8)
C8	0.069 (8)	0.067 (9)	0.068 (9)	−0.005 (7)	−0.005 (7)	−0.017 (7)
C9	0.100 (14)	0.072 (11)	0.086 (12)	0.001 (9)	−0.006 (9)	−0.017 (9)
C10	0.118 (16)	0.067 (12)	0.095 (14)	−0.030 (11)	0.023 (12)	−0.017 (9)
C11	0.086 (13)	0.111 (16)	0.093 (12)	−0.003 (12)	−0.023 (11)	−0.049 (12)
C12	0.121 (15)	0.085 (13)	0.063 (11)	0.000 (11)	0.010 (10)	−0.011 (9)
C13	0.076 (11)	0.080 (13)	0.076 (11)	−0.012 (9)	0.023 (9)	−0.015 (9)
C14	0.050 (7)	0.059 (9)	0.058 (8)	0.007 (6)	−0.003 (6)	0.014 (6)
C15	0.061 (7)	0.075 (9)	0.074 (8)	0.006 (7)	0.000 (7)	0.019 (8)
C16	0.055 (8)	0.078 (10)	0.070 (9)	−0.001 (7)	0.005 (7)	0.028 (8)
C17	0.041 (6)	0.052 (8)	0.045 (6)	0.012 (6)	0.007 (5)	0.011 (6)
C18	0.076 (9)	0.074 (10)	0.052 (8)	0.010 (7)	−0.006 (6)	0.012 (7)
C19	0.061 (9)	0.079 (10)	0.061 (8)	0.028 (7)	0.015 (7)	0.012 (7)
C20	0.045 (7)	0.088 (10)	0.070 (9)	0.003 (7)	−0.018 (6)	0.013 (8)
C21	0.060 (7)	0.062 (8)	0.051 (7)	−0.005 (7)	−0.003 (6)	0.011 (6)
C22	0.082 (11)	0.070 (11)	0.073 (10)	−0.011 (9)	−0.007 (8)	0.014 (9)
C23	0.068 (11)	0.069 (12)	0.064 (10)	0.002 (8)	0.008 (8)	0.020 (8)
C24	0.085 (11)	0.078 (11)	0.045 (8)	−0.005 (9)	0.000 (7)	0.025 (7)
C25	0.069 (10)	0.086 (11)	0.075 (9)	0.002 (10)	−0.020 (8)	0.029 (9)
C26	0.099 (13)	0.061 (11)	0.085 (12)	0.017 (10)	0.004 (10)	0.018 (9)
C27	0.033 (6)	0.051 (7)	0.044 (6)	−0.006 (5)	0.001 (5)	−0.016 (5)
C28	0.066 (8)	0.073 (9)	0.071 (8)	−0.008 (7)	−0.013 (6)	0.002 (8)
C29	0.050 (7)	0.074 (10)	0.065 (8)	−0.010 (7)	0.004 (7)	−0.025 (7)
C30	0.046 (7)	0.054 (8)	0.044 (6)	−0.006 (6)	−0.006 (5)	−0.012 (5)
C31	0.063 (8)	0.055 (8)	0.052 (7)	0.004 (7)	−0.016 (6)	−0.007 (6)
C32	0.053 (8)	0.082 (10)	0.064 (9)	−0.001 (8)	−0.003 (6)	−0.015 (8)
C33	0.075 (10)	0.073 (10)	0.057 (8)	−0.027 (8)	0.004 (7)	−0.008 (7)
C34	0.063 (8)	0.061 (9)	0.040 (7)	−0.007 (7)	−0.013 (6)	−0.001 (6)
C35	0.099 (11)	0.073 (10)	0.042 (7)	0.003 (9)	−0.006 (7)	−0.017 (7)
C36	0.082 (10)	0.084 (11)	0.070 (9)	0.002 (10)	−0.030 (8)	−0.027 (9)
C37	0.095 (11)	0.053 (9)	0.081 (10)	−0.013 (8)	−0.001 (9)	−0.015 (8)
C38	0.070 (9)	0.046 (8)	0.068 (8)	0.012 (7)	−0.009 (7)	−0.015 (7)
C39	0.077 (11)	0.069 (11)	0.066 (9)	−0.001 (8)	0.005 (8)	−0.025 (7)
C40	0.036 (6)	0.038 (7)	0.036 (6)	0.001 (5)	0.007 (5)	−0.008 (5)
C41	0.038 (6)	0.044 (7)	0.051 (6)	−0.001 (5)	0.005 (5)	−0.012 (6)
C42	0.043 (6)	0.054 (8)	0.050 (6)	0.004 (6)	0.000 (6)	−0.016 (6)
C43	0.044 (6)	0.044 (7)	0.034 (6)	0.002 (5)	0.008 (5)	−0.007 (5)
C44	0.063 (8)	0.067 (9)	0.041 (7)	0.010 (7)	0.003 (6)	−0.003 (6)
C45	0.065 (9)	0.078 (11)	0.055 (8)	0.027 (8)	−0.015 (7)	−0.012 (7)
C46	0.051 (7)	0.079 (10)	0.073 (9)	0.006 (7)	0.010 (6)	−0.022 (8)
C47	0.055 (7)	0.049 (8)	0.054 (7)	0.001 (6)	0.015 (5)	−0.006 (6)
C48	0.088 (11)	0.060 (10)	0.077 (9)	−0.017 (8)	−0.002 (8)	−0.016 (8)
C49	0.106 (13)	0.052 (10)	0.084 (11)	0.023 (9)	−0.008 (10)	−0.021 (8)
C50	0.079 (11)	0.079 (12)	0.073 (10)	−0.001 (10)	0.028 (8)	−0.028 (9)
C51	0.090 (10)	0.071 (10)	0.047 (8)	0.000 (9)	0.006 (8)	−0.015 (7)
C52	0.076 (10)	0.066 (10)	0.064 (9)	0.007 (7)	0.005 (8)	−0.016 (7)

### Geometric parameters (Å)

**Table:**

Fe1—C10	1.978 (15)	B24—H24	1.1000
Fe1—C11	1.984 (15)	B25—B29	1.695 (19)
Fe1—C12	1.999 (15)	B25—C28	1.740 (18)
Fe1—C8	2.001 (12)	B25—B30	1.763 (19)
Fe1—C4	2.020 (11)	B25—B26	1.76 (2)
Fe1—C13	2.022 (15)	B25—H25	1.1000
Fe1—C9	2.028 (15)	B26—B30	1.79 (2)
Fe1—C6	2.042 (13)	B26—B27	1.80 (2)
Fe1—C5	2.046 (14)	B26—H26	1.1000
Fe1—C7	2.051 (13)	B27—B30	1.77 (2)
Fe2—C25	2.004 (13)	B27—B28	1.80 (2)
Fe2—C22	2.006 (13)	B27—H27	1.1000
Fe2—C21	2.012 (11)	B28—B29	1.76 (2)
Fe2—C17	2.014 (10)	B28—B30	1.79 (2)
Fe2—C24	2.015 (13)	B28—H28	1.1000
Fe2—C19	2.028 (11)	B29—C28	1.712 (18)
Fe2—C20	2.032 (12)	B29—B30	1.745 (18)
Fe2—C18	2.036 (13)	B29—H29	1.1000
Fe2—C23	2.044 (15)	B30—H30	1.1000
Fe2—C26	2.054 (14)	B31—C40	1.712 (15)
Fe3—C32	1.985 (12)	B31—B32	1.726 (18)
Fe3—C31	2.022 (11)	B31—B37	1.73 (2)
Fe3—C30	2.027 (10)	B31—C41	1.738 (16)
Fe3—C37	2.029 (13)	B31—B36	1.82 (2)
Fe3—C34	2.043 (11)	B31—H31	1.1000
Fe3—C35	2.044 (13)	B32—C40	1.675 (17)
Fe3—C33	2.050 (12)	B32—B33	1.756 (19)
Fe3—C39	2.051 (14)	B32—B37	1.78 (2)
Fe3—C36	2.051 (13)	B32—B38	1.806 (18)
Fe3—C38	2.088 (12)	B32—H32	1.1000
Fe4—C45	2.001 (12)	B33—B38	1.717 (18)
Fe4—C47	2.004 (11)	B33—C40	1.725 (16)
Fe4—C46	2.019 (12)	B33—B39	1.729 (18)
Fe4—C44	2.024 (12)	B33—B34	1.789 (18)
Fe4—C49	2.034 (14)	B33—H33	1.1000
Fe4—C50	2.035 (14)	B34—C41	1.687 (18)
Fe4—C43	2.042 (9)	B34—C40	1.710 (17)
Fe4—C51	2.045 (13)	B34—B35	1.714 (19)
Fe4—C52	2.046 (15)	B34—B39	1.74 (2)
Fe4—C48	2.085 (13)	B34—H34	1.1000
O1—C3	1.400 (15)	B35—C41	1.695 (16)
O1—H1	0.8200	B35—B40	1.760 (17)
O1—H3'B	0.6617	B35—B39	1.778 (19)
O2—C16	1.31 (2)	B35—B36	1.79 (2)
O2—H2	0.8200	B35—H35	1.1000
O2—H16'	0.6567	B36—C41	1.721 (18)
O3—C29	1.354 (16)	B36—B40	1.73 (2)
O3—H3	0.8200	B36—B37	1.75 (2)
O3—H29'	0.6750	B36—H36	1.1000
O4—C42	1.369 (13)	B37—B40	1.786 (19)
O4—H4	0.8200	B37—B38	1.79 (2)
O4—H42'	0.5889	B37—H37	1.1000
O1'—C3	1.15 (5)	B38—B39	1.742 (18)
O1'—H1'	0.8200	B38—B40	1.794 (18)
O2'—C16	1.24 (2)	B38—H38	1.1000
O2'—H2'	0.8200	B39—B40	1.806 (17)
O3'—C29	1.29 (2)	B39—H39	1.1000
O3'—H3'	0.8200	B40—H40	1.1000
O4'—C42	1.33 (4)	C1—C3	1.543 (14)
O4'—H4'	0.8200	C1—C2	1.636 (16)
B1—B2	1.65 (2)	C2—H2B	1.1000
B1—C1	1.710 (17)	C3—C4	1.484 (14)
B1—B7	1.720 (19)	C3—H3B	0.9800
B1—C2	1.771 (19)	C3—H3'B	0.9800
B1—B6	1.78 (2)	C4—C8	1.398 (15)
B1—H1A	1.1000	C4—C5	1.436 (16)
B2—C1	1.650 (16)	C5—C6	1.420 (16)
B2—B3	1.687 (19)	C5—H5A	0.9300
B2—B7	1.701 (16)	C6—C7	1.365 (17)
B2—B8	1.71 (2)	C6—H6A	0.9300
B2—H2A	1.1000	C7—C8	1.385 (15)
B3—C1	1.709 (17)	C7—H7A	0.9300
B3—B9	1.75 (2)	C8—H8A	0.9300
B3—B8	1.75 (2)	C9—C13	1.311 (18)
B3—B4	1.762 (18)	C9—C10	1.38 (2)
B3—H3A	1.1000	C9—H9A	0.9300
B4—B9	1.690 (19)	C10—C11	1.41 (2)
B4—B5	1.725 (17)	C10—H10A	0.9300
B4—C1	1.741 (16)	C11—C12	1.42 (2)
B4—C2	1.810 (17)	C11—H11A	0.9300
B4—H4A	1.1000	C12—C13	1.30 (2)
B5—B9	1.71 (2)	C12—H12A	0.9300
B5—B6	1.777 (18)	C13—H13A	0.9300
B5—B10	1.79 (2)	C14—C16	1.539 (15)
B5—C2	1.795 (18)	C14—C15	1.700 (16)
B5—H5	1.1000	C15—H15A	1.1000
B6—B7	1.715 (19)	C16—C17	1.525 (15)
B6—B10	1.770 (18)	C16—H16A	0.9800
B6—C2	1.808 (17)	C16—H16'	0.9800
B6—H6	1.1000	C17—C18	1.407 (15)
B7—B10	1.778 (18)	C17—C21	1.424 (14)
B7—B8	1.80 (2)	C18—C19	1.411 (15)
B7—H7	1.1000	C18—H18A	0.9300
B8—B10	1.73 (2)	C19—C20	1.393 (15)
B8—B9	1.83 (2)	C19—H19A	0.9300
B8—H8	1.1000	C20—C21	1.396 (14)
B9—B10	1.76 (2)	C20—H20A	0.9300
B9—H9	1.1000	C21—H21A	0.9300
B10—H10	1.1000	C22—C26	1.375 (17)
B11—B12	1.66 (2)	C22—C23	1.401 (18)
B11—C14	1.706 (18)	C22—H22A	0.9300
B11—B17	1.72 (2)	C23—C24	1.337 (19)
B11—C15	1.759 (19)	C23—H23A	0.9300
B11—B16	1.81 (2)	C24—C25	1.392 (18)
B11—H11	1.1000	C24—H24A	0.9300
B12—B13	1.60 (2)	C25—C26	1.388 (18)
B12—C14	1.601 (18)	C25—H25A	0.9300
B12—B17	1.66 (2)	C26—H26A	0.9300
B12—B18	1.71 (2)	C27—C29	1.493 (13)
B12—H12	1.1000	C27—C28	1.692 (15)
B13—C14	1.642 (19)	C28—H28A	1.1000
B13—B14	1.73 (2)	C29—C30	1.479 (13)
B13—B19	1.74 (2)	C29—H29A	0.9800
B13—B18	1.76 (2)	C29—H29'	0.9800
B13—H13	1.1000	C30—C34	1.390 (14)
B14—C14	1.652 (19)	C30—C31	1.434 (13)
B14—B15	1.74 (2)	C31—C32	1.385 (15)
B14—B19	1.78 (2)	C31—H31A	0.9300
B14—C15	1.784 (19)	C32—C33	1.378 (16)
B14—H14	1.1000	C32—H32A	0.9300
B15—B19	1.77 (2)	C33—C34	1.389 (16)
B15—C15	1.777 (18)	C33—H33A	0.9300
B15—B20	1.81 (2)	C34—H34A	0.9300
B15—B16	1.84 (2)	C35—C39	1.383 (18)
B15—H15	1.1000	C35—C36	1.421 (19)
B16—B20	1.71 (2)	C35—H35A	0.9300
B16—B17	1.79 (3)	C36—C37	1.493 (18)
B16—C15	1.820 (19)	C36—H36A	0.9300
B16—H16	1.1000	C37—C38	1.424 (15)
B17—B18	1.69 (2)	C37—H37A	0.9300
B17—B20	1.73 (2)	C38—C39	1.415 (17)
B17—H17	1.1000	C38—H38A	0.9300
B18—B20	1.65 (2)	C39—H39A	0.9300
B18—B19	1.67 (2)	C40—C42	1.537 (13)
B18—H18	1.1000	C40—C41	1.657 (13)
B19—B20	1.74 (2)	C41—H41	1.1000
B19—H19	1.1000	C42—C43	1.469 (13)
B20—H20	1.1000	C42—H42	0.9800
B21—C27	1.694 (15)	C42—H42'	0.9800
B21—B26	1.717 (19)	C43—C44	1.405 (14)
B21—B22	1.736 (18)	C43—C47	1.430 (13)
B21—B25	1.758 (17)	C44—C45	1.355 (15)
B21—C28	1.766 (17)	C44—H44	0.9300
B21—H21	1.1000	C45—C46	1.360 (15)
B22—C27	1.694 (17)	C45—H45	0.9300
B22—B26	1.719 (19)	C46—C47	1.407 (15)
B22—B23	1.726 (17)	C46—H46	0.9300
B22—B27	1.798 (19)	C47—H47	0.9300
B22—H22	1.1000	C48—C52	1.457 (17)
B23—C27	1.675 (15)	C48—C49	1.468 (18)
B23—B24	1.68 (2)	C48—H48	0.9300
B23—B28	1.704 (19)	C49—C50	1.489 (19)
B23—B27	1.705 (19)	C49—H49	0.9300
B23—H23	1.1000	C50—C51	1.377 (18)
B24—B28	1.65 (2)	C50—H50	0.9300
B24—C27	1.688 (17)	C51—C52	1.379 (18)
B24—B29	1.71 (2)	C51—H51	0.9300
B24—C28	1.728 (19)	C52—H52	0.9300
